# Effect of Implant Size, Version and Rotator Cuff Tendon Preservation on the Outcome of Reverse Shoulder Arthroplasty

**DOI:** 10.7759/cureus.25741

**Published:** 2022-06-07

**Authors:** Mustafa Al Yaseen, Yat Wing Smart, Parisah Seyed-Safi, Abdelmonem H Abdelmonem, Daoud Makki, Barnes Morgan, Dilraj Sandher

**Affiliations:** 1 Trauma and Orthopaedics, West Hertfordshire Teaching NHS Trust, Watford, GBR; 2 Orthopaedic Surgery, Imperial College London, London, GBR; 3 Orthopaedics, Stockport NHS Trust, Liverpool, GBR

**Keywords:** humeral retroversion, glenosphere size, arthroplasty, reverse shoulder, rotator cuff

## Abstract

Introduction: Functional outcomes following reverse geometry shoulder arthroplasty can vary. This study assessed the effects of glenosphere size, humeral stem version, posterior rotator cuff status and subscapularis repair on patient-reported outcome and range of motion.

Methods: A consecutive series of 132 patients from two orthopaedic centres that use the same onlay system for reverse shoulder arthroplasty were reviewed over a six-year period. Outcome measures consisted of the Oxford Shoulder score (OSS) and range of motion (ROM) at one year following surgery. These were assessed against glenosphere sizes (small (36-38 mm) and large (40-42 mm)), humeral stem retroversion (less or more than 20 degrees), rotator cuff status (posterior rotator cuff present or absent) and subscapularis tendon (repaired or not) at the end of procedure.

Results: Larger glenospheres and less humeral stem retroversion yielded better ROM and OSS but this was not statistically significant. Subscapularis repair had no effect on outcomes. Preservation of posterior rotator cuff tendons improved functional outcomes. The number of tendons present at the end of procedure had a positive effect on outcome (best with two tendons and better with one compared to a completely bald humeral head).

Conclusion: Preservation of posterior rotator cuff tendons during reverse shoulder arthroplasty improves clinical outcomes unlike subscapularis repair which was found to be unnecessary. Implant size and version in reverse geometry arthroplasty have no significant effects on clinical outcome.

## Introduction

Reverse shoulder arthroplasty (RSA) and anatomical total shoulder arthroplasty are common methods used nowadays to treat shoulder joint arthritis [1]. Introduced by Neer in the 1970s, anatomical total shoulder arthroplasty has been a widely used method to treat shoulder arthritis; however, the outcome depends on the integrity of rotator cuff tendons. Therefore, RSA came into play in 1985 as a better choice with better outcome to treat rotator cuff shoulder arthropathy [2].

RSA reverses the normal shoulder anatomy in order to utilize the action of deltoid muscle to replace the deficient rotator cuff. Thus, we cannot use the ordinary methods to measure the best size of prosthesis to be used to replace the originals [2-4].

Patients’ comorbidities, psychological status and pre-operative range of motion (ROM) can all influence the outcome of RSA [5]. The effect of glenosphere size on ROM and shoulder function has been studied by computer models [6,7], physical models [8,9] and in vitro cadaveric studies [10]. The result of bony studies that did not use any soft tissue but only changing size and angle of the glenosphere proved that increasing the size can increase ROM, especially the abduction and external rotation [11]. By clearing more space for the polyethylene (PE) before impinging against the scapula (notching), lateralization of the glenoid component has shown to have a positive effect on ROM [12]. Subscapularis tendon repair has shown no effects on dislocation rate [13].

In this study, we assessed the effects of glenosphere size, humeral component version and the status of rotator cuff tendons on the outcome of RSA, in addition to whether using large or small glenospheres can change the equation.

## Materials and methods

This study consisted of a retrospective review of a consecutive series of patients with a prospective data collection of ROM and Oxford Shoulder Score (OSS) at two orthopaedics centres. All of the patients suffered from degenerative rotator cuff arthropathy to different degrees of involvement of the rotator cuff. All patients did MRI to confirm the diagnosis as part of the preparation before surgery.

The outcome measures included the range of motion and Oxford Shoulder Score pre- and postoperatively. Details from intraoperative notes included the status of the rotator cuff tendons at the end of procedure, the size and the lateralisation of the glenosphere, the version of the humeral component and whether the subscapularis tendon was repaired or not.

The two centres used two different makes of the implant but both have a similar design (onlay system).

All procedures were performed using the deltopectoral approach. Post-operative rehabilitation included early active-assisted range motion in 90 degrees of flexion and abduction, 30 degrees of external rotation for six weeks then as patient is able afterward. Patient assessment was done at six weeks, six months and one year. Oxford shoulder scores were obtained at six months and one year. The ultimate range of motion was considered to be the one that is recorded at one year and was assessed clinically using a goniometer.

The repair of the subscapularis was done whenever the soft tissue allowed, while the choice of large or small glenosphere was randomly allocated before surgery. There was no intra-operative method or way of measurement we could rely on to make that decision.

Procedures performed for fractures were excluded. The size of glenosphere was categorised as large (for glenospheres 40 mm or more) or small (for glenospheres smaller than 40 mm). Humeral component retroversion was categorised as less or more than 20 degrees. The status of rotator cuff tendons at the end of procedure was categorised as present or absent (including which tendon is still preserved). Besides, notes included whether the subscapularis tendon was repaired at the end of procedure or not (because it was not possible or because the subscapularis was absent).

Mann-Whitney U test compared the improvement in the OSS and ROM between the two groups, while subscapularis repair was assessed using Fisher’s exact test. The effect of humeral retroversion was assessed using chi-square test. Statistical analysis was carried out using the IBM SPSS version 26 (IBM Corp., Armonk, NY, USA); a p-value of less than 0.05 was considered statistically significant.

## Results

The total number of patients involved in the study was 132. The mean age of patients was 58 years (40 to 84); there were 88 males and 44 females. All the data for the 132 patients were available for one-year follow-up including OSS and ROM.

There was one patient with axillary nerve palsy who made a full recovery by two years after surgery. Two patients had acromial stress fractures that healed by 12 months postoperatively. There were no cases of dislocation or periprosthetic fractures.

**Figure 1:**
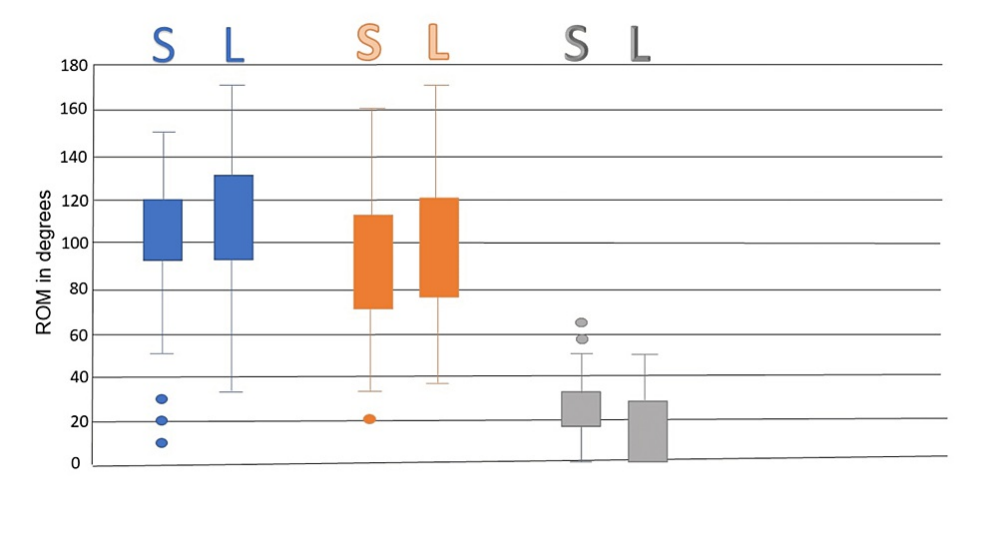
Comparison of range of motion (ROM) outcome according to the size of glenosphere. Blue: Flexion, Orange: Abduction, Grey: External Rotation, S for small glenosphere and L for large glenosphere

There were 22 patients who had the large size glenosphere and 110 patients who had the small size glenosphere. In the small glenosphere group, the median ROM at one year was 100 for flexion, 90 for abduction and 20 degrees for external rotation and the median OSS was 32. In the large glenosphere group, the median ROM at one year was 100 for flexion, 95 for abduction and 22 for external rotation and the median OSS was 36; the distribution of ROM for both groups is shown in Figure 1. The use of large glenosphere led to a better ROM and OSS; however, this was not statistically significant as shown in Table 1.

**Table 1:** Effect of glenosphere size on the outcome. * Mann-Whitney U Test ** Fisher’s Exact Test

Category	Large Glenosphere Group	Small Glenosphere Group	P Value
Total Number of Patients	22	110	---
Median Flexion in one year*	100	100	0.66
Median Abduction in one year*	95	90	0.35
Median External Rotation in one year*	22	20	0.32
Median Oxford Shoulder Score in one year*	36	32	0.29
Subscapularis Repair (%) **	15(68%)	80(72%)	0.67

We assessed the repair of subscapularis as an independent factor to evaluate its effect on external rotation and OSS in one-year follow-up. In total, the subscapularis was repaired in 95 out of 132 patients (72%) at the end of procedure. The result showed a mean OSS of 32 at one year and a median ER of 20 degrees. This was compared to a mean OSS of 32 and a median ER of 22 degrees in those who did not have the subscapularis repaired. The repair of subscapularis had no effect on OSS at one-year follow-up. However, It did restrict the external rotation by a few degrees but this was neither clinically nor statistically significant (p-value is 0.67).

Regarding the humeral retroversion, 54% of patients had a retroversion more than 20 degrees; in patients with retroversion of 20 degrees or less, the median ROM was 100 degrees for flexion, 94 degrees for abduction and 23 degrees for external rotation and the median OSS score was 35. This is compared to a median ROM of 100 for flexion, 95 for abduction and 22 for external rotation in patients with a retroversion of more than 20 degrees and a median OSS of 32. A chi-square test was used to assess the correlation between retroversion and ROM and OSS; the correlation was statistically non-significant as shown in Table 2. Humeral component retroversion had no impact on ROM and OSS in this study as shown in Table 2.

**Table 2:** Effect of humeral retroversion on outcome * Chi-Square Test

Category	Humeral retroversion <= 20 degrees	Humeral Retroversion >20 degrees	P Value
Total number of patients	71	61	---
Mean Flexion in one year *	100	100	0.66
Mean Abduction in one year *	94	95	0.32
Mean External Rotation in one year *	23	22	0.65
Mean Oxford Shoulder Score in one year *	35	32	0.91

The status of rotator cuff tendons at the end of the procedure was divided into four categories: 1- All present but degenerative supraspinatus; 2- Only Supraspinatus is absent; 3- Only teres minor is present 4- All torn (bald head). Results showed that the number of intact tendons positively affects the functional outcome. The mean flexion, abduction and ER were significantly better when we compared each of categories 1, 2 and 3 to category 4. Teres minor was found to be the most important as its presence alone yielded as good function as that from intact tendons in categories 1 and 2 (no statistically significant difference between means of ROM parameters between categories 1 and 3 and also between 2 and 3). Results are summarised in Table 3.

**Table 3:** Effect of rotator cuff status on the outcome. OSS: Oxford Shoulder Score

Rotator cuff status	Number of patients (percentage)	Mean Flexion	Mean Abduction	Mean Ex Rotation	OSS
Category 1: teres ok, infra ok, supra present but (pasta or degenerative)	62(47%)	125.6	100.7	30	36
Category 2: teres ok, infra ok, supra-absent	32(24%)	128	100.3	28	34
Category 3: teres ok, infra torn	12(9%)	123.3	95	25	34
Category 4: All torn	26(20%)	103.7	88	20	32

## Discussion

In theory, larger glenospheres lead to a better ROM and more stability compared to smaller ones [14]. However, the downside for that is more deltoid tension and risk of stress acromial fracture. Other factors such as the status of rotator cuff tendons, pre-operative ROM and other patient-related factors can affect the functional outcome of RSA [15,16]; in this study, we focused primarily on the effects of glenosphere size, humeral component version and the rotator cuff status on the outcome.

Daniel et al. studied the effect of the glenosphere size on the joint reaction force and ROM; they proved that the larger the size the better the ROM and more joint reaction force but yet didn’t assess the soft tissue factors [4]. The glenosphere size did not have a significant effect on the ROM or the functional score; despite the subtle differences in the range of flexion and external rotation in favour of larger glenospheres, this difference was neither clinically nor statistically significant (p-value: 0.35 and 0.32 respectively). Such a result can be attributed to the fact that the outcome is a result of interplay of many other factors; one of them is the rotator cuff status.

Giles et al. investigated the impact of cuff repair on RSA outcome, they investigated the effect of both the cuff repair and the glenosphere lateralization in vitro. Interestingly, they concluded that cuff repair antagonizes the RSA function [17]. Likewise, Erikson et al. studied the impact of rotator cuff repair prior to RSA and concluded that this did not offer any significant improvement [18]. On the contrary, our study showed that intact rotator cuff tendons yield better function and that out of all four tendons, teres minor was found to be the most important. This could be explained by the potential preservation of external rotation while the arm is abducted, a function that is provided by teres minor and that is required in many daily tasks. We believe that preservation of as many rotator cuff tendons as possible during RSA should theoretically offer a better outcome, but this needs to be studied further through randomised trials.

Daniel et al, studied the effect of humeral retroversion on ROM in RSA and concluded that more retroversion conferred a better ROM [19]; in their study, the variation in retroversion was quite vast (-20 to +40 degrees) and that is difference between the lowest and highest two retroversion recorded in their series of patients. In our study, retroversion of humeral implant had no impact on range of motion or function, and this confirms the findings of other studies [20,21]. However, it is important to note that the variation of retroversion in our series of patients was between 5 and 30 degrees and, when compared to the study by Daniel et al. [19], we can conclude that it is possible that retroversion within 40 degrees (within normal variants) does not have an impact on ROM and function.

Edwards et al. studied the effect of subscapularis repair on RSA dislocation rate; they found there is significant relation between the repair and the dislocation rate, the more the repair the less the dislocation rate [13]. Unlike our study which showed of 28% of cases with no subscapularis repair done, none have a recorded dislocation.

Limitations

The follow-up time for the patients was only one year, which is not that long of a period for shoulder surgeries. For the aim of getting a large number, more than one surgeon was involved in the study which might put some surgical variation in managing soft tissue during the surgery. However, the technique and the system were unified.

## Conclusions

Preservation of rotator cuff tendons during reverse shoulder arthroplasty improves clinical outcomes while subscapularis repair is found to be unnecessary. Implant size and version in reverse geometry arthroplasty have no significant effects on clinical outcome.
